# An inhibitor of cholesterol absorption displays anti-myeloma activity by targeting the JAK2-STAT3 signaling pathway

**DOI:** 10.18632/oncotarget.12265

**Published:** 2016-09-26

**Authors:** Xin Xu, Kunkun Han, Jingyu Zhu, Hongwu Mao, Xu Lin, Zubin Zhang, Biyin Cao, Yuanying Zeng, Xinliang Mao

**Affiliations:** ^1^ Jiangsu Key Laboratory of Translational Research and Therapy for Neuro-Psycho-Diseases, Department of Pharmacology, College of Pharmaceutical Sciences, Soochow University, Suzhou, China; ^2^ School of Pharmaceutical Sciences, Jiangnan University, Wuxi, China; ^3^ Department of Oncology, Suzhou Municipal Hospital East Campus, Suzhou, China; ^4^ Jiangsu Key Laboratory of Preventive and Translational Medicine for Geriatric Diseases, Soochow University, Suzhou, China

**Keywords:** JAK2, STAT3, multiple myeloma, SC09

## Abstract

The activated JAK2-STAT3 signaling pathway is a high risk factor for multiple myeloma (MM), a fatal malignancy of plasma cells. In the present study, SC09, a potential inhibitor of cholesterol absorption, was identified in a STAT3-targeted drug screen. SC09 suppressed the activation of STAT3 in a time-course and concentration-dependent manner but did not affect its family members STAT1 and STAT5. SC09 inhibited STAT3 transcriptional activity and downregulated the expression of STAT3-regulated genes. Further studies showed that SC09 selectively inhibited JAK2 activation but not other kinases including c-Src, ERK, p38 and mTOR that are all associated with STAT3 activation. Moreover, SC09 obviously induced MM cell death *in vitro* and delayed MM tumor growth *in vivo*. SC09-induced MM cell death was dependent on the endogenous STAT3 status, and this effect could be attenuated by enforced expression of STAT3. All the results collectively indicated that SC09 blocks the JAK2-STAT3 signaling thus displaying anti-MM activity. Given its well tolerance and anti-MM potency, SC09 is credited for further investigation as a promising drug for MM treatment.

## INTRODUCTION

Multiple myeloma (MM) is a class of fatal and progressive hematological malignancy derived from plasma cells, the cells that produce antibodies. MM is the second most blood cancers and accounts for 2% of all cancer-associated death worldwide [1]. Last decades have witnessed the progress in MM pathophysiology and in the development of drug discovery, however, there is no cure for this specific disease [2]. Development of novel drugs is urgent.

Molecularly targeted drug discovery is a promising strategy for the precision treatment of various cancers. During the last decade, a panel of molecularly targeted anti-cancer drugs have been marketed and have been demonstrated to be effective in both complete remission and progression-free survival. It has been demonstrated that MM is a genetic disease featured with dysregulation of some molecular events, of which the transcription factor STAT3 is of interest and plays a prominent role in myelomagenesis [3, 4]. STAT3 is highly expressed in more than 63% of CD138^+^ bone marrow cells of MM patients [5]. By modulating important pro-survival genes, STAT3 promotes MM cell proliferation, survival and adhesion, and protects cells from drug-induced cell apoptosis [6]. STAT3 also contributes to drug resistance. A recent study found that suppression of the STAT3 signaling pathway by the tight junction protein 1 increases proteasome inhibitor sensitivity in MM cells [4]. In addition to drug sensitivity and resistance, STAT3 also predicts poor prognosis of MM patients. A sequencing screen over 142 untreated MM patients revealed that STAT3-mutation contributes a statistically significantly shortened progression-free survival and overall survival in these patients [7]. All the above findings provide a solid rationale for MM treatment by targeting the STAT3 signaling. Indeed, inhibition of STAT3 activity by small molecule compounds leads to MM cell apoptosis [8]. In the present study, we found the inhibitor of cholesterol absorption SC09 displays anti-MM activity by suppressing the JAK2-STAT3 signaling pathway.

## RESULTS

### SC09 inhibits STAT3 activation in MM cells

**Figure 1:**
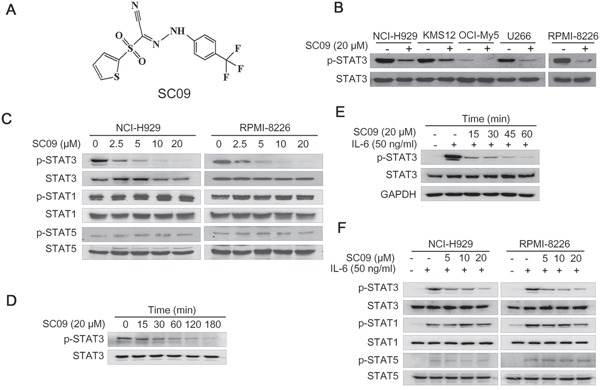
SC09 inhibits STAT3 activation **A.** The chemical structure of SC09. **B.** MM cell lines were treated with SC09 overnight, followed by immunoblotting against p-STAT3 and total STAT3. **C.** NCI-H929 and RPMI-8226 cells were treated with SC09 at indicated concentrations, followed by analysis of STAT1, STAT3 and STAT5 activation. **D.** RPMI-8226 cells were treated with SC09 at 20 μM for different periods, followed by immunoblotting assay against STAT3 activation. **E.** OCI-My5 cells were starved overnight, followed by treatment with SC09 at 20 μM for different time points before IL-6 (50 ng/ml) stimulation for 20 min. **F.** After starved overnight, NCI-H929 and RPMI-8226 cells were treated with SC09 at indicated concentrations for 4 h before IL-6 (50 ng/ml) stimulation for 20 min, followed by analysis of STAT1, STAT3 and STAT5 phosphorylation.

To find out novel inhibitors that disrupt the STAT3 signaling for MM treatment, we designed a STAT3-luciferase-based high throughput screen. Using this screening system, several candidates of STAT3 inhibitors were identified [8]. In the present study, we focused on the small molecule compound SC09 (Figure 1A), a reported inhibitor of cholesterol absorption (International Patent Publication Number: WO2007/008529A2). In this study, SC09 was preliminarily found to reduce the expression of luciferase under control of STAT3-recognition elements by the high throughput screen, which suggested that SC09 was probably an inhibitor of the STAT3 signaling pathway. To verify this hypothesis, a panel of MM cell lines were treated with SC09 overnight, followed by immunoblotting assay. As shown in Figure 1B, SC09 decreased STAT3 phosphorylation at Tyr705, an activated form of STAT3, in all cell lines examined. And this effect was concentration-dependent (Figure 1C). To find out whether SC09 could inhibit STAT3 activation in a short-time period, RPMI-8226 cells were incubated with SC09 from 15 to 180 min, followed by evaluation of STAT3 phosphorylation at Tyr705. As shown in Figure 1D, SC09 suppressed STAT3 activation as early as in 15 min, and more than 80% was suppressed within 60 min, which thus suggested that SC09 rapidly inhibited STAT3 activation. Next, OCI-My5 cells were starved overnight followed by SC09 treatment and IL-6 stimulation. The results showed that IL-6, a key trigger of STAT3 signaling, markedly activated STAT3, but it was suppressed by SC09 in a time-dependent manner (Figure 1E). And this effect was found in RPMI-8226 and NCI-H929 cells in a concentration-dependent manner (Figure 1F). Because STAT3 is one of the 6 members in the STAT superfamily, of which STAT1 and STAT5 are also involved in cell proliferation. Therefore, we further evaluated the STAT1 and STAT5 activation levels after SC09 treatment. As shown in Figure 1F, SC09 markedly suppressed the activation of STAT3, but it did not show any effects on STAT1 or STAT5. These findings collectively suggested that SC09 selectively inhibits STAT3 activation.

### SC09 inhibits the transcriptional activity of STAT3 in MM cells

**Figure 2:**
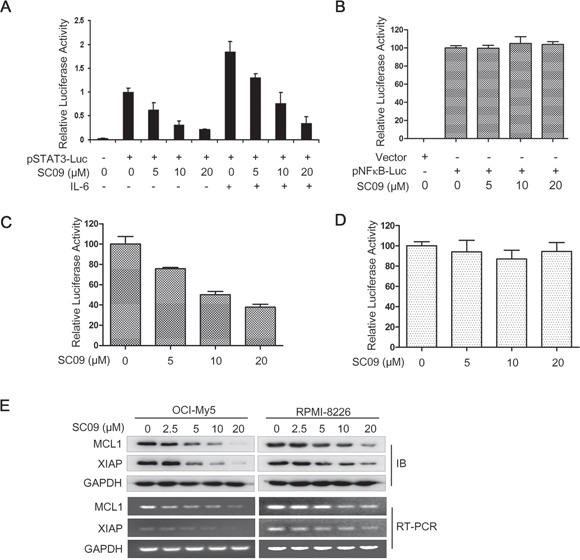
SC09 inhibits STAT3 transcriptional activity **A.** HeLa cells were transfected with vector or pSTAT3-Luc by Lipofectamine2000 and maintained for 24 h before being treated with SC09 at indicated concentrations for continued 12 h and IL-6 (50 ng/ml) stimulation for 20 min. Luciferase assays were performed. **B-D.** HeLa cells were transfected with different luciferase reporters driven by NF-κB response elements (B), cyclin D2 promoter containing (C) or lacking (D) STAT3 response element for 24 h, respectively, and all cells were treated with SC09 at indicated concentrations for 12 h before being subjected to luciferase assay. **E.** OCI-My5 and RPMI-8226 cells were treated with SC09 at indicated concentrations for 24 h before being harvested for immunoblotting and RT-PCR against MCL1 and XIAP, respectively.

Because STAT3 is a transcription factor and the above studies demonstrated that SC09 is a STAT3 inhibitor, we wondered whether SC09 suppressed the transcriptional activity of STAT3. To this end, we made a construct of STAT3 recognition element (SRE)-driving firefly luciferase as the reporter and transfected HeLa cells, followed by SC09 treatment. As shown in Figure 2A, SC09 inhibited luciferase activity driven by SRE. When cells were stimulated with IL-6, SC09 remained this inhibitory activity (Figure 2A). In contrast, SC09 failed to inhibit luciferase activity driven by the recognition element of NF-κB, another important transcription factor (Figure 2B). To confirm this effect, a luciferase construct driven by the cyclin D2 promoter containing or lacking a STAT3 recognition element was applied for the study. SC09 suppressed the luciferase activity driven by the CCND2 promoter containing a STAT3 recognition element (Figure 2C) but not by the promoter lacking a STAT3 recognition element (Figure 2D). These findings thus demonstrated that SC09 suppressed STAT3 transcriptional activity. It is well known that STAT3 modulates the expression of a broad spectrum of genes, including XIAP and MCL1 [9]. Therefore, we next measured the expression of these two genes. As shown in Figure 2E, SC09 downregulated the expression of these genes at both the protein and mRNA levels. Therefore, SC09 inhibits STAT3 transcriptional activity.

### SC09 inhibits the JAK2-STAT3 signaling pathway in MM cells

**Figure 3:**
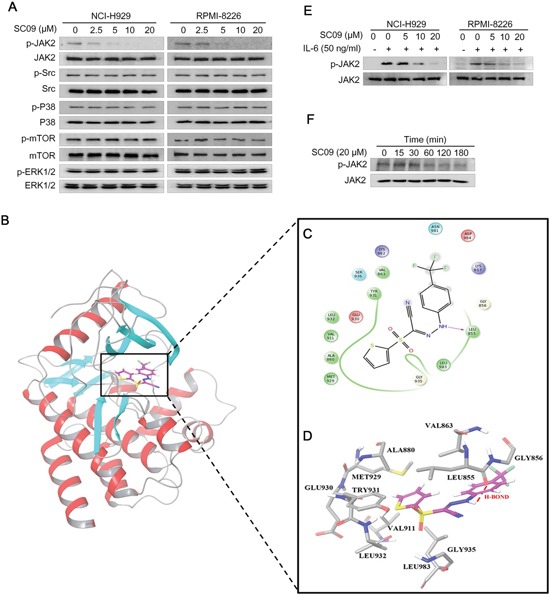
SC09 suppresses JAK2 phosphorylation **A.** NCI-H929 and RPMI-8226 cells were treated with SC09 at indicated concentrations overnight, followed immunoblotting assay against kinases as indicated. **B.** SC09 was docked into the ATP-binding pocket of JAK2 by computer modeling. **C.** SC09 formed extensive van der Waals interactions with the hydrophobic residues of JAK2. **D.** SC09 formed strong and stable hydrogen bonds with Leu855 of JAK2 (in Red). **E.** NCI-H929 and RPMI-8226 cells were starved overnight before SC09 treatment at indicated concentrations for 4 h and IL-6 (50 ng/ml) stimulation for 20 min, followed by analysis of JAK2 activation. **F.** RPMI-8226 cells were treated with SC09 at 20 μM for different time points, followed by immunoblotting assay against phosphorylated JAK2.

The above studies concluded that SC09 inhibits STAT3 activation and transcriptional activity. However, as a transcription factor, STAT3 is regulated by several other factors, typically including a panel of kinases, such as JAK2, c-Src, mTOR, and mitogen-activated protein kinases (p38 and ERK) [10]. To understand the inhibitory mechanism of SC09 on STAT3 activation, we evaluated the activation of these kinases in the presence of SC09 in MM cell lines RPMI-8226 and NCI-H929. As shown in Figure 3A, SC09 selectively inhibited JAK2 phosphorylation but had no effects on other kinases tested in the assay. Therefore, we wondered whether JAK2 was the acting target of SC09. To verify this hypothesis, we performed a computer docking analysis. As shown in Figures 3B–D, SC09 was well docked into the active pocket of JAK2 in which SC09 formed extensive van der Waals interactions with the hydrophobic residues of JAK2 (Figure 3C) and formed strong and stable hydrogen bond (red line in Figure 3D) with Leu855 of JAK2. These results indicated that SC09 strongly interacted with JAK2 and led to the inhibition of JAK2 activity.

We next measured the JAK2 activation level in MM cells. In both NCI-H929 and RPMI-8226 cells, SC09 inhibited JAK2 phosphorylation in a concentration-dependent manner in the presence of IL-6, the major stimulator of JAK2-STAT3 signaling pathway (Figure 3E). More importantly, SC09 also suppressed endogenous JAK2 activation at a level similar to the inhibition of STAT3 (Figure 3F, Figure 1D). We also measured the inhibitory efficacy of SC09 on the activity of JAK2 and its family members. JAK2 is a non-receptor kinase of the Janus kinase family including JAK1, JAK2, JAK3 and TYK2 [11]. In the cell-free enzymatic assay, SC09 was found to prefer to inhibit JAK2 activity. Of these 4 enzymes, the IC_50_ of SC09 on JAK2 was 8.88 μM, more than 10 times lower than those to other kinases (IC_50_s to JAK1, JAK3 and TYK2 were 152.7, 138.9 and 111.5 μM, respectively). We also measured the inhibition of SC09 on c-Src and the IC_50_ was 276.2 μM. Notably, this result to c-Src was consistent to the immunoblotting assay as shown in Figure 3A and SC09 had no effects on c-Src activation. Therefore, we could conclude that SC09 preferred to inhibit JAK2 activity and presented itself as an inhibitor of the JAK2-STAT3 signaling pathway.

### SC09 induces MM cell apoptosis in association with STAT3 activation

**Figure 4:**
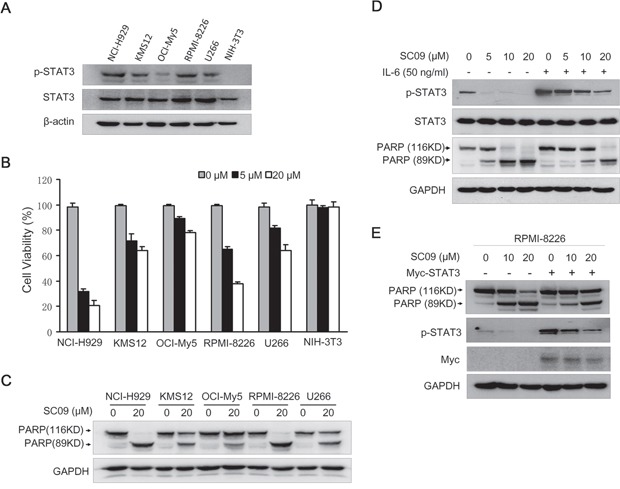
SC09 induces MM cell apoptosis by suppressing STAT3 activation **A.** The expression profile of p-STAT3 in MM cell lines and NIH-3T3 cells. **B.** MM and NIH-3T3 cells were treated with SC09 at indicated concentrations for 24 h, and cell viability was measured by trypan blue staining. **C.** MM cell lines were treated with SC09 for 24 h followed by an immunoblotting assay to evaluate the cleavage of PARP. **D.** RPMI-8226 cells were treated with SC09 at indicated concentrations with or without 50 ng/ml of IL-6 for 24 h. Cells were harvested and prepared for immunoblotting against specific antibodies. **E.** Myc-STAT3 plasmids or empty vectors were transfected into RPMI-8226 for 24 h, followed by SC09 treatment at 10 or 20 μM for 24 h. Cells were harvested and prepared for immunoblotting against PARP, p-STAT3 and Myc.

SC09 had been demonstrated to be a potent JAK2-STAT3 inhibitor, we therefore wondered whether SC09 induced MM cell apoptosis. We firstly evaluated whether MM cell sensitivity to SC09 was associated with endogenous expression level of activated STAT3. As shown Figure 4A, all MM cells harbored aberrantly active STAT3 (p-STAT3), while NIH-3T3 cells did not express active STAT3 (p-STAT3), which was consistent with previous findings [12]. In the subsequent analysis on cell proliferation, the results showed that the endogenous STAT3 activation determined cell sensitivity to SC09. As shown in Figure 4B, cells such as NCI-H929 and RPMI-8226 expressing a high level of p-STAT3 were sensitive, but NIH-3T3 cells lacking p-STAT3 were resistant to SC09. OCI-My5 with less activated STAT3 was less sensitive to SC09 than H929 and RPMI-8226 (Figure 4B). These results were confirmed in the cell apoptosis as evidenced with the cleavage levels of PARP (Figure 4C). All these findings pointed to that SC09 sensitivity was dependent on the STAT3 activation level. To verify this hypothesis, RPMI-8226 cells were treated with SC09 in the presence or absence of IL-6, followed by immunoblotting assay to evaluate cell apoptosis. As shown in Figure 4D, when STAT3 was over-activated by IL-6, SC09-induced PARP cleavage was partly abolished. To confirm this finding, RPMI-8226 cells with enforced expression of STAT3 were applied for SC09 sensitivity assay. As shown in Figure 4E, STAT3 phosphorylation was increased upon the transfection, along with decreased PARP cleavage. All the above studies thus collectively demonstrated that SC09 targeted STAT3 signaling and it induced MM cell apoptosis in association with endogenous activated STAT3 level.

### SC09 delays MM tumor growth in nude mice without overt toxicity

**Figure 5:**
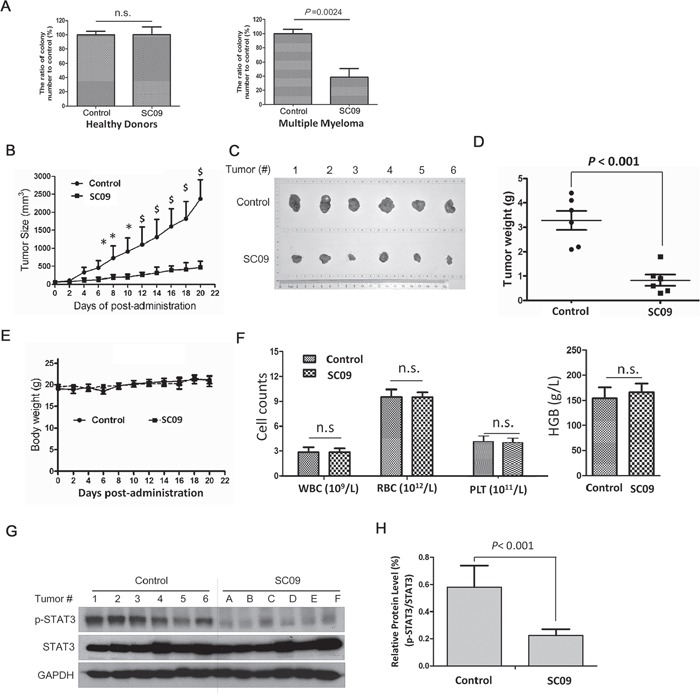
SC09 delays MM tumor growth with well tolerance *in vivo* **A.** Mononuclear cells isolated from bone marrow collected from healthy donors or MM patients were treated with SC09 (10 μM) for 24 h before being plated in triplicate in MethoCult GF H4434 medium and cultured for 3 weeks before counting the colony forming units. **B.** The curves of tumor sizes over the experiment. *, *p* < 0.05; $, *p* < 0.01. **C.** The tumor tissues were excised at the end of the experiment. **D.** The weight of the excised tumor tissues was measured. **E.** The curve of body weights over the experiment period. **F.** White blood cells (WBC), red blood cells (RBC), platelets (PLT) and hemoglobin (HGB) were measured at the end of the experiment. **G.** The excised tumor tissues were prepared for immunoblotting against p-STAT3, STAT3 and GAPDH. **H.** Statistical analysis for p-STAT3/STAT3 of Figure G.

SC09 had been demonstrated to induce MM cell apoptosis, to further understand its anti-MM activity, bone marrow cells isolated from healthy donors and MM patients were subjected to colony forming assay in the presence or absence of SC09. The results showed that there were no difference in colony counts from healthy donors treated with SC09, however, SC09 significantly decreased the colony forming units from bone marrow cells of MM patients (Figure 5A). Therefore, SC09 probably preferred to inhibit MM progenitors or stem cells.

Next, we evaluated tumor growth from a human MM xenograft in nude mice after SC09 treatment. As shown in Figure 5B, oral administration of SC09 at a dose of 30 mg/kg markedly decreased tumor growth in one week, and the average of tumor volumes was reduced up to 78% compared to the vehicle control at the end of the experiment (20 d). The tumor sizes and weights presented in a similar manner as the volume on the last day (Figures 5C and D). However, SC09 did not affect mice body weights throughout the experimental period (Figure 5E). Blood analysis revealed that SC09 did not markedly change the counts and measurements of the red blood cells, white blood cells, platelets and hemoglobin (Figure 5F). These results implicated that SC09 was probably a minimal toxic agent. Because SC09 was an STAT3 inhibitor, we wondered whether STAT3 activation was suppressed in tumor tissues. To this end, tumor species were excised from mice at the end of the experiment and subjected to p-STAT3 measurement. As shown in Figures 5G and H, SC09 markedly inhibited STAT3 phosphorylation. This assay thus demonstrated that SC09 delayed MM tumor growth by targeting STAT3 signaling.

### SC09 enhances MM cell apoptosis induced by doxorubicin

**Figure 6:**
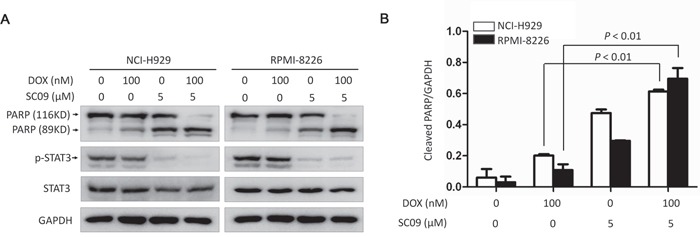
SC09 enhances DOX-induced cell apoptosis in MM **A.** NCI-H929 and RPMI-8226 cells were treated with Doxorubicin (DOX) and/or SC09 at indicated concentrations for 24 h, followed by immunoblotting assay against PARP and GAPDH. **B.** Statistical analysis of PARP cleavage from A.

Various studies have shown that over-activated STAT3 contributes chemoresistance to anti-MM agents, such as doxorubicin (DOX) [13, 14], while downregulation of STAT3 can enhance tumoricidal effects [15, 16]. Therefore, we wondered whether SC09 as a STAT3 inhibitor could enhance cytotoxicity of DOX against MM. To this end, MM cell lines NCI-H929 and RPMI-8226 were treated SC09 alone or in combination with DOX, followed by immunoblotting assay for apoptosis. As shown in Figures 6A and B, SC09 significantly enhanced MM cell death induced by DOX in terms of PARP cleavage. DOX at 100 nM induced 20% PARP cleavage in NCI-H929 cells and SC09 induced 50% PARP cleavage at 5 μM, but more than 95% PARP was cleaved when combined with 5 μM of SC09 and 100 nM of DOX. In RPMI-8226 cells, similar tendency was observed in PARP cleavage (Figures 6A and B). Because PARP cleavage is a common marker of apoptosis, this finding suggested that SC09 enhanced DOX-induced MM apoptosis and probably overcomes DOX chemoresistance.

## DISCUSSION

The above studies identified SC09 as a novel JAK2-STAT3 inhibitor from a high throughput screen using STAT3 recognition element-driving firefly luciferase as the reporter.

Because of its significance in carcinogenesis and poor clinical outcomes, STAT3 has been developed as an ideal drug target for various cancer treatment [17–19]. Currently various inhibitors have been identified, of which OPB-51602 has been evaluated in Phase I clinical trial for the treatment of patients with relapsed/refractory hematological malignancies, including acute myeloid leukemia (AML), non-Hodgkin's lymphoma, MM, or chronic myeloid leukemia [20]. However, in addition to most common side effects such as nausea, peripheral sensory neuropathy, and diarrhea, grade 3 or 4 drug-related adverse events were also found in a high frequency, including neutropenia (20%), leukopenia (15%), lymphopenia (10%), and thrombocytopenia (10%) [20]. Relatively, our compound did not show significant changes in the measurement of red blood cells, white blood cells, platelets and hemoglobin. In addition, SC09 does not affect the body weights of model mice during the experimental course although it markedly decreased tumor growth. In the experimental study with primary patients' bone marrow cells, SC09 prefers to inhibit clonogenic growth of MM bone marrow cells but it does not affect colony forming in healthy donors. Therefore, SC09 is probably a relative safe agent for the treatment of MM.

To execute its activity in gene transcription, STAT3 is usually activated by either non-receptor kinases such as c-Src and JAKs [21] or mitogen-activated protein kinases (MAPK) such as ERK and p38 [22]. Our study suggested that SC09 specifically suppresses the JAK2-STAT3 signaling pathway. There are four members in the JAK family including JAK1, JAK2, JAK3 and Tyk2, but enzymatic analyses showed that SC09 specifically inhibits JAK2. Of note, SC09 does not inhibit c-Src and other kinases including AKT, mTOR, ERK and p38, although these kinases also increase STAT3 activation. Of note, there are six members in the STAT family of which STAT1, STAT3 and STAT5 are highly associated with cancer [23], but SC09 specifically inhibits STAT3 activation thus inducing cancer cell death. Moreover, SC09 inhibits transcriptional modularity of STAT3 but not another nuclear transcription factor NF-κB although both cross-talk in some cancers. Therefore, SC09 specifically inhibits the JAK2-STAT3 activation and this selective inhibition is important in anti-cancer drugs because it will reduce toxicity or adverse effects and increases well tolerance. In contrast, non-selective agents, such as cytotoxic agents (e.g. plant alkaloids and anti-tumor antibiotics), raise a high level of toxicity in healthy tissues and organs.

It is known that both constitutive activation and cytokine-induced activation of the JAK2-STAT3 signaling pathway are also critical in MM chemoresistance. IL-6, the key cytokine stimulus of the JAK2-STAT3, is overexpressed in MM cells and contributes resistance to major anti-MM agents [24], while blockade of the IL-6 signaling transduction by administration of IL-6-specific monoclonal antibody benefits patients with relapsed or refractory MM by overcoming certain resistance [25]. Overactivated STAT3 also contributes to resistance in MM but downregulation of STAT3 overcomes such resistance. Similar to some other STAT3 inhibitors, SC09 also overcomes chemoresistance in MM cells and enhances therapeutic efficacy of other known anti-MM drugs by suppressing both IL-6-induced and endogenous activity of STAT3. DOX is a cytotoxic anti-tumor antibiotics and a major anti-MM agent among the most used anti-MM regimens. Our study showed that SC09 synergistically acts on MM cells with DOX, which is clinically significant because of the emerging resistance to DOX [26].

Notably, a filed patent shows that SC09 is an inhibitor of cholesterol absorption because SC09 is claimed to suppress the transcription of ABCA1, a cholesterol transporter. Cholesterol is an integral part of the plasma membrane of animal cells to maintain their structural integrity and modulate their fluidity. Cholesterol is reported to be associated with survival and proliferation of MM cells that require more cholesterol than normal cells [27]. In consistence with this finding, LDL-cholesterol suppresses MM cell apoptosis in culture [28] and blockade of the cholesterol transport by an anti-cancer agent JNJ-26854165 induces MM cell apoptosis [27]. If SC09 indeed inhibits cholesterol absorption, it will be of interest because the JAK2-STAT3 pathway is found to promote ABCA1 expression [29]. Therefore, it is possible that SC09 as an inhibitor of the JAK2-STAT3 inhibits cholesterol absorption by downregulating ABCA1 expression. It has been reported that several anti-cholesterol agents display potent anti-cancer activity such as Statins [30]. However, whether SC09 blocks cholesterol absorption should be further examined.

In summary, the present study found that SC09 is a selective JAK2-STAT3 inhibitor and displays potent anti-MM activity in both *in vitro* and *in vivo* models. Taken into consideration with its potential inhibition in cholesterol absorption, it will be of interest to further investigate its anti-MM activity in association with its anti-STAT3 and anti-cholesterol activity.

## MATERIALS AND METHODS

### Cell culture and regents

All cell lines NCI-H929, KMS12, OCI-My5, RPMI-8226 and U266 were kindly provided by Dr. Aaron Schimmer from Ontario Cancer Institute, Toronto, Canada. Cell culture was described previously [8]. Primary bone marrow samples were collected from the Department of Hematology, the First Affiliated Hospital of Soochow University, and the assay was approved by the Institutional Review Board of Soochow University. Informed consent was obtained in accordance with the Declaration of Helsinki. Mono-nuclear cells were isolated by Lympholyte® Cell Separation (Cedarlane, Canada) [31]. SC09 was purchased from Maybridge Chemicals (Trevillett, UK). Interleukein-6 (IL-6) was purchased from Cell Signaling Technology (Danvers, MA).

### Immunoblotting analysis

Whole cell lysates were prepared for immunoblotting as described previously [32, 33]. Specific primary antibodies against PARP, Caspase 3, MCL1, XIAP, p-STAT3(Tyr705), STAT3, p-STAT1(Tyr701), STAT1, p-STAT5(Tyr694), STAT5, JAK2, p-Src(Ser17), c-Src, p-P38(Thr180/Tyr182), P38, p-ERK1/2(Thr202/Tyr204), ERK1/2, p-mTOR(Ser2448) and mTOR were purchased from Cell Signaling Technology (Danvers, MA); a specific primary antibody against p-JAK2(Tyr1007/Tyr1008) was purchased from Santa Cruz Biotechnology, Inc. (Santa Cruz, CA); anti-Flag and anti-Myc antibodies were purchased from Medical & Biological Laboratories (Tokyo, Japan). GAPDH and β-actin were purchased from Abgent (Suzhou, China). Anti–mouse immunoglobulin G (IgG) and anti–rabbit IgG horseradish peroxidase conjugated antibody were purchased from R&D Systems (Minneapolis, MN).

### Cell viability assay

Myeloma cells were dispensed in 96-well plates at a density of 1.5×10^4^ cells per well and treated with increasing concentrations of SC09 for 24 h, The viable cells were evaluated by trypan blue exclusion staining as described previously [8].

### Plasmids construction and gene transfection

The human transcription factor STAT3 cDNA was cloned into pcDNA3.1 vector with a Myc tag generously as described previously [8]. The regulatory sequences of cyclin D2 promoter with or without a STAT3 response element was inserted into pGL4 vector (Promega Corporation, Madison, WI, USA) as previously described [34]. A STAT3-driving luciferase construct (pSTAT3-luc) and a NF-κB luciferase construct (pNF-κB-Luc) were purchased from Beyotime Biotechnology Institute (Nantong, China) [8]. Plasmids were transiently transfected into HeLa or RPMI-8226 cells by Lipofectamine^®^ 2000 (Invitrogen) according to the manufacturer's instruction.

### Molecular docking

The molecular docking analysis was performed as described previously [8]. The structure of SC09 was constructed in Schrodinger (version 09) and then preprocessed with the *LigPrep* module. The structure of JAK2 (PDB ID: 4ZIM) was used as the initial structure. All crystallographic water molecules were removed, hydrogen atoms were added, and the structure was submitted to restrained minimization to relieve steric clashes using the OPLS2005 force field within the *Protein Preparation Wizard* in Schrodinger. The minimization was terminated when the root-mean-square deviation (RMSD) reached a maximum value of 0.3 Å. For the grid generation and ligand docking procedures, the default settings were used and the *Glide* module in the Schrodinger was employed for molecular docking.

### Luciferase assay

HeLa cells were transfected with pSTAT3-Luc, CCND2-Luc, pNF-κB-Luc or empty vector by Lipofectamine^®^ 2000 (Invitrogen) according to the manufacturer's instruction. Twenty-four hours later, cells were incubated with SC09 for 12 h, and cells were prepared for luciferase assay using Dual-Luciferase^®^ Reporter Assay System (Promega, Madison, WI, USA) as described previously [32, 34].

### Reverse transcription-polymerase chain reaction (RT-PCR)

OCI-My5 and RPMI-8226 cells were treated with SC09 for 24 h, and then cells were harvested and prepared for RT-PCR as described previously [35]. The primers used were as follows: MCL1, forward 5’-GCGACGGCGTAACAAACT-3’ and reverse 5’-ATTCCTGATGCCACCTTCTAG-3’; XIAP, forward 5’-AACCTTGTGATCGTGCCT-3’ and reverse 5’-CCAATCAGTTAGCCCTCC-3’; GAPDH, forward 5’-AGTCCACTGGCGTCTTCA-3’ and reverse 5’-CTCCGACGCCTGCTTCACCA-3’.

### Protein kinase activity assay

The effects of SC09 on purified kinases including c-Src, JAk1, JAK2, JAK3 and Tyk2 were evaluated using the HotSpot technology and it was performed at Reaction Biology Corp. (Malvern, PA, USA) as described previously [36]. In short, assays contained a peptide substrate, purified recombinant human protein kinases to be tested, and gamma-labeled ATP, magnesium ion. Radioactive phosphorylated product was measured and quantitated via a scintillation counter. Compounds were tested in a 10-dose IC_50_ mode with 3-fold serial dilutions starting at 300 μM. Staurosporine that inhibits many protein kinases through the prevention of ATP binding to the active pocket was used as a positive control.

### Colony forming assay

A colony forming unit assay was performed to evaluate clonogenic growth of bone marrow cells from primary MM patients and healthy donors as described previously [8]. Briefly, bone marrow cells (6.25 × 10^5^/mL) treated with SC09 or buffer control for 24 h were washed and plated in triplicate in standard MethoCult GF H4434 medium (StemCell Technologies, Vancouver, BC) as described previously [8]. The number of colonies containing 20 or more cells was counted for statistical analysis.

### Myeloma xenograft study

The human myeloma xenograft model was established in nude mice (Shanghai SLAC Laboratory Animal Co., Ltd., Shanghai, China) with RPMI-8226 cell line as described previously [8]. When tumors were palpable, mice were randomly divided into two groups. One group was orally received SC09 (30 mg/kg) in PBS containing 10% Tween 80 and 10% DMSO, and another was given vehicle for continued 20 d. At the same time, tumor sizes and body weights were measured every other day. At the end of the experiment, tumors were excised for immunoblotting and blood samples were collected and subject to hematological analysis. This animal study was approved by the Review Board of Animal Care and Use of Soochow University.

### Statistical analysis

Student's *t* test was used for comparisons of two groups in the *in vitro* studies. All statistical tests were two-sided, and when *p* < 0.05, the difference was considered to be statistically significant.
